# Meetings of Societies

**Published:** 1906-06

**Authors:** 


### MEETINGS OF SOCIETIES.

### Bristol Medico-Chirurgical Society.

A Correction.—In the report of the meeting of the Society on February 14th, 1906, Mr. W. Roger Williams is credited with having stated that "in the last fifty years the mortality of phthisis had decreased threefold." Mr. Williams informs us that what he actually said was, that "during the last sixty-five years the phthisis mortality had declined threefold, viz. from 380 per 100,000 living in 1838 to 119 in 1903."

*March 14th, 1906.*

Mr. John Dacre, President, in the Chair.

An address on **The Position of Pathology with regard to Clinical Diagnosis** was given by A. W. Mayo Robson, D.Sc., F.R.C.S., Emeritus Professor of Surgery in the University of Leeds.

The address was highly appreciated by the large number of members and visitors who attended the meeting, and subsequently a cordial vote of thanks was given to Prof. Mayo Robson.

---

*April 11th, 1906.*

Mr. John Dacre, President, in the Chair.

Dr. J. A. Nixon showed a specimen of **Calcareous Supra-renal Capsules.**

Mr. Munro Smith read notes on a case of **Thrombosis of Mesenteric Vessels.** The patient, a middle-aged man, was seized suddenly with abdominal pain and sickness. The symptoms ran a sub-acute course for more than two weeks, and then became more marked. The abdomen was opened, and three feet of small intestine found to be dark red, but not œdematous. Resection was not attempted. The patient died three weeks after the initial symptoms. The whole of the superior mesenteric artery and the mesenteric veins were found to be thrombosed at the autopsy. No cardiac, pulmonary, or arterial disease could be discovered.—Dr. Bertram Rogers alluded to a case he had reported of thrombosis of the mesenteric veins in which the patient was a boy. The symptoms had lasted two or three months, and included abdominal pain, distension and ascites. There had not been obstruction of the bowels nor melæna.—Dr. Stack asked if there had been evidence as to whether the thrombosis was due to embolism or to the contrary.—Mr. Munro Smith replied that there was no definite sign that an embolism was the cause. No blood had been passed *per rectum.*

Dr. J. Michell Clarke read a paper on "**The Urinary Nitrogenous Excretion in Bright's Disease as a Guide to Treatment.**"

Dr. G. Parker gave an account of a case of **Splenic Leukæmia with the symptoms of Gout.** The leukæmia could be traced to a malarial origin, and was of a severe type with 220,000 white cells, 25 per cent. of myelocytes and a huge spleen. Under treatment by cacodylate of sodium and the X-rays the patient recovered sufficiently to return to work for a time. Acute attacks of arthritis commencing in the left knee, but affecting also the great toe and the other joints, with redness and swelling and pain, commenced about the same time as the leukæmia. Deposits of urate of sodium formed in the ear and on both index fingers. The urine had throughout shown albumen, granular and other casts were noted; 270 grains of urea and 3 to 6 grains of purin bodies in the twenty-four hours had been found during a quiescent period. The interest of the case lay in the fact that in leukæmia, although there is an enormous increase of endogenous uric acid, the symptoms of gout are practically unknown. Garrod and Roberts think that this is because the uric acid is freely excreted by the healthy kidneys. Here, the kidneys being impaired, uratic deposits in the joints took place, *i.e.* the great excess of uric acid plus the retention produced uratosis, a fact which is not without importance as to some theories of gout.

---

*May 10th, 1906.*

Mr. JOHN DACRE, President, in the Chair.

Dr. BARCLAY BARON showed a case of **Excision of portion of the Larynx for Epithelioma** in September, 1904, with no recurrence and good voice.

Dr. J. O. SYMES showed a case of **Arthritis with large spleen** in an adult.

Mr. T. CARWARDINE showed (*a*) a **Charcot's Knee,** (*b*) a **Diseased Elbow,** (*c*) a case of **Recurrent Intussusception** at six and nine months of age.

Dr. J. MICHELL CLARKE showed a case of "**Peroneal or Neurotic Muscular Atrophy.**"

Mr. J. TAYLOR showed **Skiagrams** of rare and interesting clinical conditions.

Dr. J. A. NIXON showed (*a*) a case of **Lichen Scrofulosorum,** (*b*) two cases of **Herpes Ophthalmicus.**

Dr. WATSON WILLIAMS demonstrated models illustrating the **Clinical Anatomy of the Nose and Accessory Sinuses.**

Dr. KENNETH WILLS showed cases of **Tænia Circinata.**

Dr. FORTESCUE-BRICKDALE showed a case of **Tremor of unilateral distribution.**

Dr. WALTER G. CHASE (of Boston, U.S.A.) gave a demonstration by Cinematograph of **Epilepsy and other Clinical Phenomena of Nervous Diseases.**

J. LACY FIRTH.
H. F. MOLE, *Hon. Sec.*

---

### Newport Medical Society.

At the Newport and Monmouthshire Hospital on Thursday, May 17th, 1906, Sir Thomas Myles, ex-President of the Royal College of Surgeons in Ireland, delivered an address on the value of the X-rays to the general practitioner. Mr. W. F. Green, President of the Society, occupied the chair, and there was a large audience, including many of the medical men of Bristol, Cardiff, and the surrounding districts.

The Lecturer specially directed the attention of his audience to a series of lantern slides illustrating the importance of expert X-ray examination in the diagnosis of injuries in the neighbourhood of the shoulder-joint. The pictures demonstrated clearly that many accidents which were formerly considered to be of the nature of sprains or bruises are actually associated with damage to bony structures, and the lecturer expressed his opinion that fractures of the great tuberosity are more common than is usually believed. Excellent examples of X-ray work were exhibited, which showed:—(*a*) **Fractures of the anatomical neck** (1) without impaction, (2) with impaction, (3) with impaction and separation of the tuberosities, (4) with rotation of the head into a horizontal position, (5) with impaction and comminution, (6) with dislocation of the head; (*b*) **Fractures of the surgical neck,** (1) showing a large spicule of bone, (2) transverse without displacement, (3) transverse with displacement, (4) with impaction, (5) with impaction from a case in which diagnosis of the condition without radiography was impossible, (6) without any deformity, (7) old-standing with a large ring of callus, (8) old ununited, (9) with dislocation of the head; (*c*) **Dislocations,** (1) simple sub-coracoid, (2) sub-coracoid with doubtful fracture of the anatomical neck, (3) sub-coracoid with tearing of the tuberosities, (4) sub-glenoid with tearing of the tuberosities; (*d*) **Separation of the epiphysis.** A number of further slides were then thrown on the screen, including illustrations of such rare conditions as reversed Colles's fracture, dislocation of the ulna from the radius at the lower end, Bennet's fracture of the thumb, transverse fracture of the tibia without displacement and compression fracture of the tibia.

Mr. R. Lane Joynt, Surgeon to the Meath Hospital, Dublin, who had carried out the X-ray work for the demonstration, was present and assisted the lecturer.

On the motion of Dr. Garrod Thomas, seconded by Mr. J. Lynn Thomas, C.B., and supported by Mr. J. Paul Bush, C.M.G., and others, a hearty vote of thanks was passed to Sir Thomas Myles and Mr. Lane Joynt.

Sir Thomas Myles, in replying, thanked the audience for the cordiality of his reception, and expressed his appreciation of the expert manner in which the lantern was manipulated by Mr. Geo. R. Thompson.

---

